# Real-time PCR for direct aptamer quantification on functionalized graphene surfaces

**DOI:** 10.1038/s41598-019-55892-3

**Published:** 2019-12-17

**Authors:** Viviane C. F. dos Santos, Nathalie B. F. Almeida, Thiago A. S. L. de Sousa, Eduardo N. D. Araujo, Antero S. R. de Andrade, Flávio Plentz

**Affiliations:** 1 Departamento de Física, ICEx, Universidade Federal de Minas Gerais, Avenida Presidente Antônio Carlos 6627, Belo Horizonte, CEP 31270-901 Brazil; 2 Centro de Desenvolvimento da Tecnologia Nuclear (CDTN), Avenida Presidente Antônio Carlos 6627, Campus Pampulha da Universidade Federal de Minas Gerais, Belo Horizonte, CEP 31270-901 Brazil; 3 Departamento de Física, CCE, Universidade Federal de Viçosa, Avenida Peter Henry Rolfs, s/n, Viçosa, CEP 36570-900 Brazil

**Keywords:** Biosensors, DNA and RNA

## Abstract

In this study, we develop a real-time PCR strategy to directly detect and quantify DNA aptamers on functionalized graphene surfaces using a *Staphylococcus aureus* aptamer (SA20) as demonstration case. We show that real-time PCR allowed aptamer quantification in the range of 0.05 fg to 2.5 ng. Using this quantitative technique, it was possible to determine that graphene functionalization with amino modified SA20 (preceded by a graphene surface modification with thionine) was much more efficient than the process using SA20 with a pyrene modification. We also demonstrated that the functionalization methods investigated were selective to graphene as compared to bare silicon dioxide surfaces. The precise quantification of aptamers immobilized on graphene surface was performed for the first time by molecular biology techniques, introducing a novel methodology of wide application.

## Introduction

DNA (deoxyribonucleic acid) aptamers are single strand oligonucleotides that presents high affinity and specificity to their binders^1^. In comparison with traditional ligands, such as antibodies, aptamers present some advantages. They are chemically stable, cost-effective, more resistant to pH and temperature variations, and are more flexible in the design of their structures^2^. Due to these characteristics, they have great potential as sensing components in diagnostic and detection assays. Biosensor platforms based on DNA aptamers are more stable for storage and transport than the antibodies counterparts^2^.

Nanotechnology applications in medicine have been showing great results in diverse fields, like diagnostics, for instance^3–5^. A large number of publications investigated the use of nanomaterials, such as graphene and carbon nanotubes, on biosensors development^6–8^.

Nanomaterials hold great potential as biosensors devices by affording for lower detection limits, with better selectivity than traditional assays. Graphene is a bidimensional lattice of carbon atoms arranged in an alveolar structure^9–11^. As a conductive two-dimensional material, every single carbon atom is in direct contact with the environment and can readily respond to the environment fluctuations caused by external agents. Good examples are the ability to even detect single molecules and single micro-organisms^12,13^. This makes graphene an ideal candidate for sensing applications, for instance as a transducer in biosensors devices^14^.

Due to its planar geometry, several molecules can be directly attached to the graphene surface. This makes it very suitable for biochemical sensors since surface modification to make it selective is essential for this application. Graphene-based biosensors functionalized with aptamers have already been demonstrated to detect a wide range of targets, such as cancer molecules^15^, *Staphylococcus aureus*^16^, DNA^17^, glucose^18^, bacteria on tooth enamel^13^ and immunoglobulin E^19,20^. For all applications, a key factor is to transform the graphene surface into a selective but sensitive surface. This can only be accomplished by means of highly efficient and reproducible functionalization strategies.

In the context of biosensing, graphene grown by chemical vapor deposition (CVD) is one of the most promising materials for large-scale applications in this field^18,21,22^. Currently, the evaluation of aptamer incorporation on the graphene surface is performed by qualitative techniques such as atomic force microscopy (AFM) and fluorescence microscopy. The analysis of aptamer incorporation by fluorescence microscopy is very difficult to perform due to the fluorophore’s emissions quench by the underlying graphene^23^. In the specific case of AFM, a detailed evaluation of such incorporations demands high resolution scanning, which is time-consuming and limited to small areas. Additionally, CVD graphene usually contains intrinsic defects and chemical impurities, which may lead to tip artifacts and misinterpretations in AFM topography imaging^24–26^.

In particular, biomolecules association with graphene are generally assigned by charge transfer between these compounds. In this sense, Raman spectroscopy is a technique sensitive to doping and, in principle, it could be explored do quantify the incorporation of aptamers on graphene. Charge transfer between graphene and molecular adsorbates are monitored by shifts and/or broadening/narrowing of the G and 2D bands of graphene^27^. To use this tool for quantitative analysis of aptamer incorporation, for example, the quantity of aptamers attached on the graphene surface must be previously known in order to correlate any changes in the G and 2D bands of graphene with this quantity. Nowadays, this calibration method is lacking.

So far, the real effectiveness of the surface functionalization with aptamers can only be determined by the final biosensor response to the target biomolecules^16^. Therefore, the lack of prior quantitative knowledge about the surface coverage by aptamer makes difficult a more precise study, encompassing a correlation between the biosensor device final performance and the surface functionalization quality. This difficulty impairs and delays the optimization of functionalization procedures and protocols.

Real-time quantitative Polymerase Chain Reaction (qPCR) is a well-known technique applied for DNA detection and quantification^28^. A considerable number of biosensors are based on DNA aptamers, so it is possible, in principle, to use qPCR to detect these molecules on the functionalized surfaces. qPCR is a widespread tool able to amplify DNA and, consequently, it can detect very small amounts of this biomolecule. With this technique, it is possible to work with very small amounts of starting material and analyze a very limited amount of raw sample. By using a standard curve, it is possible to calculate the initial quantity of the target DNA with high sensitivity and precision taking into account the exponential amplification phase of the reaction. Due to its sensitivity, reliability and simplicity qPCR has become the most widely used technique for quantifying DNA^29^. Despite all its potential for DNA detection, this assay has not been used, as far as we know, to detect and quantify the immobilization of DNA based aptamers in nanomaterials, especially graphene. In this sense, these techniques have a great potential to be explored in the analysis of aptamer-functionalized nanomaterials.

In this work was developed a qPCR strategy to detect and quantify aptamers in functionalized graphene surfaces. Using this novel quantitative methodology was possible to compare directly the efficiency of different kinds of functionalization in graphene with unprecedented simplicity and accuracy. With the increasing interest in DNA aptamers-based biosensors this work provides a novel and efficient tool for the development of these nanobiodevices.

## Methods

### Graphene and silicon dioxide samples

The graphene substrates used in this work consist of CVD graphene on top of a 285–300 nm-thick SiO_2_ layer, grown by dry oxidation on a highly-doped p-type silicon substrate (Si/SiO_2_). The graphene substrates were purchased from either Graphenea Inc. or Grolltex Inc. The pristine Si/SiO_2_ substrates were purchased from Waferpro LLC. The actual 2.5 × 2.5 mm, for graphene samples and 1.5 × 1.5 mm for silicon dioxide samples used in the work were diced from the substrates.

### Aptamers and Primers

The *S. aureus* binding aptamer SA20^30^ was modified in the 5′ end with a 3C spacer followed by a pyrene cap phosphoramidite (SA20-pyrene) and in the 5′ end with a 6C spacer followed by an amine moiety (SA20-amino). The SA20 aptamer sequence is:

5′-GCAATGGTACGGTACTTCCGCGCCCTCTCACGTGGCACTCAGAGTGCC

GGAAGTTCTGCGTTATCAAAAGTGCACGCTACTTTGCTAA -3′.

Primers for qPCR were designed using the Primer 3 software (http://frodo.wi.mit.edu/)

The primer sequences were:

SA 20 forward: 5′- GCAATGGTACGGTACTTCC -3′ and

SA 20 reverse: 5′- AACTTCCGGCACTCTGA -3′.

### Functionalization of the graphene and silicon sample**s**

Graphene and pristine silicon dioxide samples functionalization with SA20-pyrene were performed by overnight incubation in a wet atmosphere with a drop of 1.0 μM SA20-pyrene solution dispersed in 1.0 mM PBS (phosphate buffered saline- 0.0001 M phosphate buffer, 0.000027 M potassium chloride and 0.00137 M sodium chloride), pH7.4. After incubation, the samples were first washed with 1.0 mM PBS for 15 minutes and then were let immersed again in 1.0 mM PBS overnight. Lastly, the samples were dried with N_2_ gas before performing PCR assays. The reason to keep the samples in 1.0 mM PBS overnight was to allow sufficient time for desorption of excess molecules that did not bind to the exposed surfaces.

Graphene and pristine silicon dioxide samples functionalization with SA20-amino was performed by at first binding thionine into the graphene samples surfaces. Thionine has already been used to bind biomolecules to carbon nanotubes^31^.

The thionine functionalization step was performed by 15 minutes incubation with a drop of 1.0 mM thionine chloride (Santa Cruz Biotechnology) solution followed by washing with 1.0 mM PBS for 10 minutes and overnight incubation in a wet atmosphere with a drop of a solution of 1.0 μM SA20-amino aptamer dispersed in 1.0 mM PBS, pH7.4. As in the pyrene-based method, the samples were washed with 1.0 mM PBS for 15 minutes and kept immersed in 1.0 mM PBS overnight. The last step was to blow dry the samples with N_2_ before performing PCR assays.

The materials functionalization with the SA20 and SA20-amino aptamers performed without previous surface modifications were carried exactly as described above after the surface modification steps.

### Conventional PCR reactions

Conventional PCR reactions were carried out in a Veriti Thermal Cycler (Applied Biosystems, Foster City, CA) in order to assess primers performance before qPCR. The assays were performed in 60 µl reaction, which contained 15 U of Taq DNA polymerase (Ludwig Biotec, Alvorada, RS) in the presence of 10X buffer (6 µl), 2.5 mM of MgCl_2_ and 1.0 µM of each primer (SA forward and reverse). The functionalized graphene samples were placed directly in PCR tubes.

The cycling conditions were performed as follows: after an initial denaturation step of 2 min at 94 °C, 25 cycles were performed for 45 s at 94 °C, 30 s at 60 °C, and 1 min at 72 °C. A final extension step for 10 min at 72 °C was used.

At the end of PCR cycles, the aptamers presence was checked on a standard 2% agarose gel stained with ethidium bromide. A total of 8 functionalized graphene samples were tested by conventional PCR (4 functionalized with SA20-pyrene and 4 with thionine and SA20-amino). The negative template (NTC, without DNA) and positive (with 10 ng of SA20 aptamer) controls were performed.

### Real-Time quantitative PCR (qPCR)

Reactions were carried out in a Step One Real-Time PCR System (Applied Biosystems, Foster City, CA) using the Power Up SYBR Green Master Mix (Applied Biosystems, Foster City, CA). The assays were performed in 20 µl final volume of reaction containing 10 µl of the Master Mix and 250 nM of each primer (SA20 forward and reverse). Graphene and silicon dioxide samples were placed directly on the PCR plate wells. After an initial enzyme UDG activation at 50 °C for 2 min and an enzyme dual-lock DNA polymerase activation at 95 °C for 2 min, 40 amplification cycles were performed using 95 °C for 15 s for denaturation and 60 °C for 1 min for annealing, extension, and fluorescence acquisition.

A standard curve with SA20 aptamer was performed using concentrations ranging from 0.00005 fg to 5.0 ng with a dilution factor of 1:10. In all assays, we used a positive control with 0.5 ng of the SA20 aptamer to evaluate the reaction reliability. In all of these experiments a non-functionalized graphene or silicon dioxide slice was included on all the wells of the standard curve and positive controls in order to circumvent and take into account any possible graphene or silicon dioxide interference in the essays, such as in the fluorescence acquisition. This is important to guarantee the experiments reproducibility and reliability. A negative template control (NTC), which contain all the reaction components but does not have any DNA (template), was also included in all q PCR assays. All samples, standards and controls were processed in triplicate

For each qPCR assay, a total of 12 functionalized graphene or functionalized pristine silicon dioxide samples were tested. These devices were divided into 3 groups containing four samples each that were functionalized at different occasions.

In order to obtain the number of aptamers per square centimeter, PCR results obtained in nanograms were converted to grams and this value was divided by molecular weight of the corresponding aptamer divided by Avogadro constant. After that the results (number of aptamers) were divided by the sample area in cm^−2^. The actual area covered by graphene in the graphene samples was measured by optical microscopy using as a guideline the contrast difference between graphene covered x uncovered sample surface. We used only graphene samples with more than 75% of covered area.

### Statistical analysis

Data normality was tested using the D’Agostino-Pearson omnibus test. Statistical analysis were performed using the One way ANOVA with Bonferroni´s multiple comparison test for the comparison of the SA20-pyrene and SA20-amino linked on graphene and silicon surfaces. Student’s t-test was used for all other analyses by means of the software package GraphPad Prism 5.0 (Graph-Pad Software, San Diego, CA, USA).

## Results and Discussion

The SA-20 aptamer immobilization on graphene was achieved in this work by non-covalent functionalization, in order to better preserve the inherent properties of pristine graphene. For comparison purposes, and in order to look for the best strategy of aptamer incorporation, two different methods of functionalization were used to attach the aptamers to graphene. In the direct functionalization approach, pyrenil moieties (pyrene cap phosphoramidite) were previously introduced at the 5´end of the aptamers (SA20-pyrene). The SA20 aptamer modified with this pyrene functional group, has an affinity to the graphene π orbitals and can be directly attached onto the graphene surface via π-stacking^32^.

In the indirect approach, amine moieties are introduced at the 5´end of the SA-20 aptamer. This amino-functionalized aptamer (SA20-amino) can now bind to surfaces having chemical affinity to the amine functional groups. To allow the SA20-amine aptamers binding to the graphene, a previous functionalization step promoting thionine molecules incorporation into the graphene surface was included. The thionine molecules then act as an intermediate binding layer between the SA20-amino and the graphene surface.

Thionine has a planar aromatic structure that allows strong interaction with the surface of graphene sheets through synergistic non-covalent charge-transfer and π-stacking^33^, leaving a large amount of hydrophilic amino groups (NH_2_) available for interaction with other molecules. In a recent work^34^, we have demonstrated that, contrary to what was previously believed, thionine molecules do not lie flat on the graphene but are vertically attached to the surface. In this same work, we also demonstrated that the thionine bounded onto the graphene surface undergoes a structural transition, driven by the amount of surface coverage, were a preferential alignment of domains along graphene crystallographic directions takes place.

qPCR was developed in early 1990s for nucleic acids detection and quantification; it is widely used in almost all fields of biomedical research, agriculture, food and environment sciences^28,35–37^.

In our approach, in order to monitor DNA amplification during qPCR assays, the SYBR Green dye was employed. This dye is a fluorescent nucleic acid stain that binds double-stranded DNA molecules by intercalating between the DNA bases. The reason for choosing this dye for quantitative PCR is because the fluorescence can be measured at the end of each amplification cycle to determine, relatively or absolutely, how much DNA was amplified.

**Figure 1:**
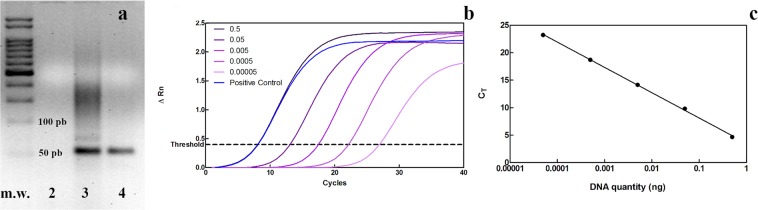
Representative results obtained using qPCR primer sets in conventional and qPCR. **(a)** 2% typical agarose gel stained with ethidium bromide showing the amplification of the sequence of approximately 56 bp, referent of SA20 aptamer at the end of the PCR process. Molecular weight (M.W.), negative template control (NTC, lane 2), positive control (lane 3) and a SA20-pyrene functionalized graphene (lane 4). Full-length unmodified gel is presented in Supplementary Figure 1. **(b)** Amplification curves of real-time PCR (using 10-fold serial dilutions of SA20 aptamer ranging from 0.5 ng to 0.00005 ng) representing the amplification of each point in the standard curve and the amplification of the positive control (with 0.5 ng of SA20). **(c)** Standard curve in which the Ct values were plotted against the template quantity. These curves (**b** and **c**) are representative of 10 independent experiments, all presenting similar results.

PCR assays yielded a single product of 56 bp (the expected amplification size by using the primer set), as it can be observed in the lanes of positive control and of SA20-pyrene functionalized graphene. Negative template control did not presented any amplification (Fig. 1a). The qPCR standard curves were optimized using several SA20 aptamer dilutions, and the optimized reaction conditions were obtained using from 0.00005 to 0.5 ng of aptamer (Fig. 1b). These standard curves were generated by plotting the SA20 aptamer quantity log *versus* the corresponding Ct (cycle threshold) value. The Ct corresponds to the PCR cycle in which a particular sample emits enough fluorescence to reach the threshold above the background fluorescence. The linear regression determination coefficient (*R*^2^) was 0.999 in all performed assays as represented on Fig. 1c, indicating linear responses in the SA20 aptamer detection.

The minimum and maximum qPCR limits of detection were determined considering the linear part of the curve of serially diluted SA20 aptamer. Quantities ranging from 0.05 fg to 2.5 ng of SA20 could be detect by qPCR, independent of 5´end modification. The same range of detection was also obtained for qPCR using pristine Si/SiO_2_ samples, instead of graphene samples. These findings highlight the qPCR sensibility and flexibility for detection of a wide range of aptamer quantities. Moreover, these limits of detection are in accordance with the expected number of aptamer molecules to be found on the functionalized graphene or silicon surface.

After qPCR standardization, this methodology was used to quantify the number of SA20-pyrene and SA20-amino molecules that were retained on graphene surface after the functionalization protocols. All of qPCR data showed R^2^ of 0.999. Twelve graphene samples were functionalized with thionine and SA20-amino (indirect functionalization), and other twelve directly with SA20-pyrene (direct functionalization). In Table 1 was present the obtained results. The mean value of SA20-pyrene molecules surface coverage was 277 × 10^8^ cm^−2^, whereas for the SA20-amino this mean value was 2385 × 10^8^ cm^−2^, indicating one order of magnitude higher surface coverage. In addition, Table 1 also shows that the number of molecules per square centimeter detected in all samples for the same functionalization type was similar, which is characterized by a low standard deviation, showing the functionalization process reproducibility. According to these results, the functionalization with SA20-amino, using thionine as an intermediate molecule, was considerably more efficient than the SA20-pyrene functionalization of the graphene surface.

**Table 1:** Number of SA20-amino and SA20-pyrene aptamer molecules per square centimeter retained on graphene and silicon dioxide surface in three independent experiments. For the functionalization with SA20-amino, the material surfaces were previous treated with thionine.

Aptamer	Material	Experiment 1		Experiment 2		Experiment 3		Mean * (×10^8^)
		Samples (×10^8^)	Mean (×10^8^)	Samples (×10^8^)	Mean (×10^8^)	Samples (×10^8^)	Mean (×10^8^)	
SA20-amino and Thionine	Graphene	2830	3623 ± 539	3240	3160 ± 494	2990	2385 ± 408	3056 ± 690
		3740		3820		2100		
		3930		2700		2210		
		3990		2880		2240		
	Silicon dioxide	20	16 ± 10	1	5 ± 4	3	14 ± 8	12 ± 9
		19		9		20		
		24		3		19		
		1		7		14		
SA20- pyrene	Graphene	260	197 ± 46	146	160 ± 36	309	277 ± 25	212 ± 61
		150		185		277		
		192		116		271		
		185		195		249		
	Silicon dioxide	2	3 ± 1	4	5 ± 1	6	4 ± 2	4 ± 1
		3		6		3		
		2		5		3		
		4		5		3		

*Mean and standard deviation of the three experiments.

Although this results demonstrate that qPCR is an effective method for quantitative detection of functionalization it is still important to further demonstrate that it can be applied to readily identify the specificity and effectiveness of functionalization protocols.

The issue to be investigated is if the SA20 really attach preferably onto the graphene surface, which is responsible for the sensing activity, rather on the silicon dioxide. To address this central question, additional experiments were performed using pristine Si/SiO_2_ as substrates for functionalization with both SA20-amino and SA20-pyrene aptamers. Our goal is to directly access and compare the affinity of these aptamers for graphene and for silicon dioxide.

In Table 1 was shown that the SA20-amino aptamer has a mean surface coverage area of 12 × 10^8^ cm^−2^ whereas the SA20-pyrene aptamer shows a mean coverage of 4 × 10^8^ cm^−2^ on silicon dioxide surface. This result confirms that the aptamers affinity was appreciably greater for graphene than for silicon dioxide. The SA20-amino (in association with thionine) and SA20-pyrene bond to graphene via π-π stacking. The absence of this mechanism may be a plausible reason for the low affinity of these aptamers with the silicon dioxide, in comparison with graphene.

**Figure 2:**
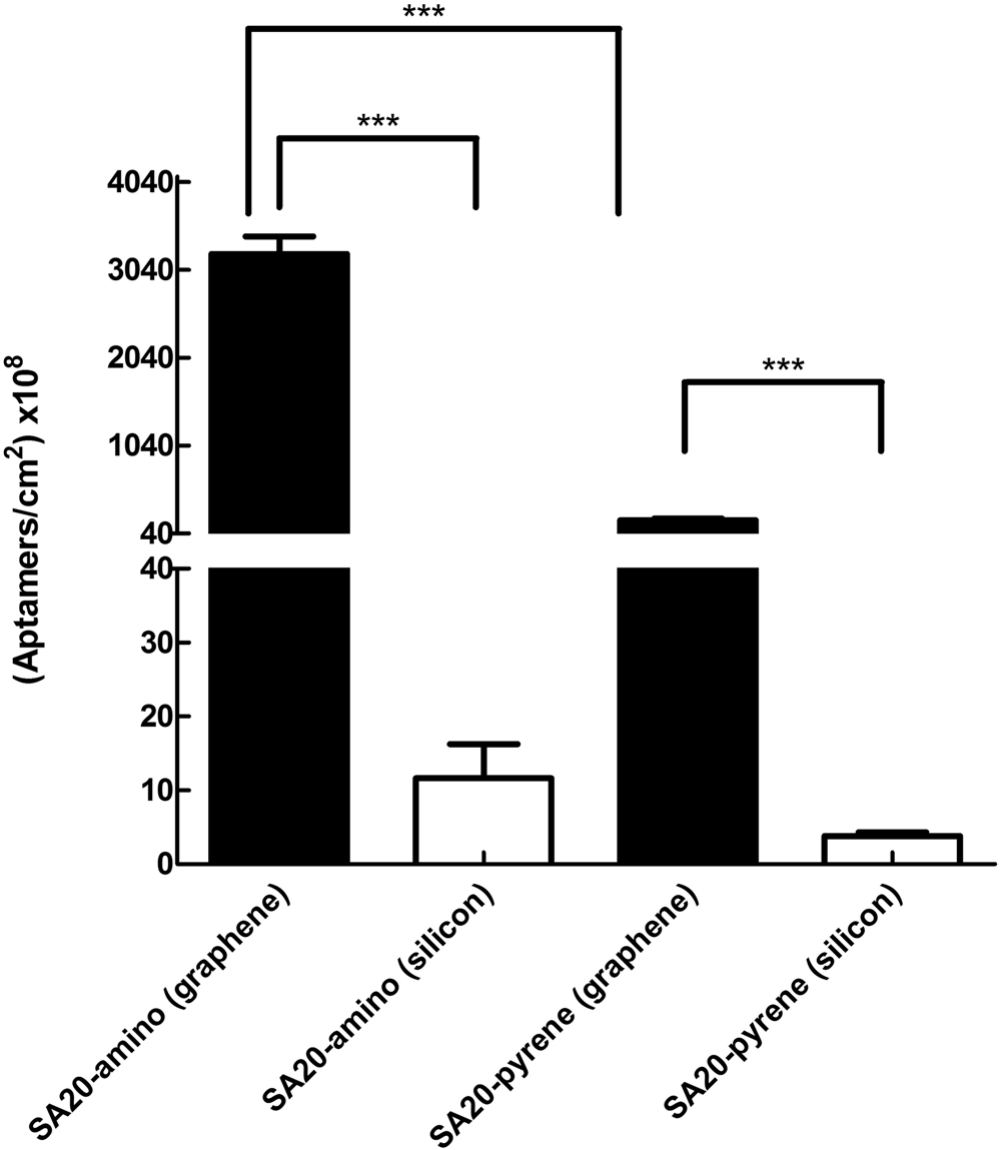
Quantification of SA20-amino and SA20-pyrene retained on graphene and silicon dioxide surface after functionalization. Graphene and Silicon dioxide samples were functionalized with thionine and SA20- amino or with SA20-pyrene and subjected to absolute quantification by qPCR. Data represent the mean ± SEM of three independent experiments, using 4 samples in each one. ***P < 0.0001. The F value for this one way ANOVA test was 248.5.

The SA20-amino, in association with graphene thionine functionalization, and SA20-pyrene bond to graphene via π-stacking could be, for the first time, directly and quantitatively compared and proved to be efficient surface modification strategies. In Fig. 2, all the results were summarized.

Although it was clear that SA20-pyrene and SA20-amino conjugated to thionine have much higher affinity to graphene than to silicon dioxide, a significant number of molecules were retained on silicon dioxide surface. In order to further explore our methodology, and also evaluate the aptamers binding without any functional group modification to either SiO_2_ or graphene surfaces, a set of experiments employing unmodified aptamers were conducted.

The mean value of SA20 aptamers surface coverage on the silicon dioxide surface was 59 × 10^8^ cm^−2^, whereas on graphene surface this number was 57 × 10^8^ cm^−2^ (Table 2). There is no statistical difference between the number of molecules in graphene or in silicon dioxide surfaces when they are functionalized with aptamers without any modification.

**Table 2:** Number of SA20 without any modification and SA20-amino aptamer molecules per square centimeter retained on graphene and silicon dioxide surface in three independent experiments. In these experiments for the functionalization with SA20-amino, the material surfaces were not previous treated with thionine.

Aptamer	Material	Experiment 1		Experiment 2		Experiment 3		Mean * (×10^8^)
		Samples (×10^8^)	Mean (×10^8^)	Samples (×10^8^)	Mean (×10^8^)	Samples (×10^8^)	Mean (×10^8^)	
SA20	Graphene	73	58 ± 19	53	56 ± 5	64	57 ± 15	57 ± 13
		42		52		50		
		76		57		40		
		40		63		74		
	Silicon dioxide	27	109 ± 135	24	39 ± 27	50	31 ± 18	59 ± 81
		41		74		16		
		310		12		14		
		56		45		42		
SA20- amino without Thionine	Graphene	5	14 ± 7	24	16 ± 10	6	9 ± 6	13 ± 8
		12		24		2		
		18		3		14		
		20		14		13		
	Silicon dioxide	44	22 ± 18	1	2 ± 1	3	12 ± 8	12 ± 13
		19		1		20		
		24		3		15		
		1		1		8		

*Mean and standard deviation of the three experiments.

Since amine is a very polar group, we could imagine that amine modified aptamers could have also a large affinity to bare graphene surfaces, what was not the case. To stress the fundamental role played by surface functionalization, SA20-amino was also used to functionalize graphene samples without previous functionalization with thionine.

In Table 2 it can be observed that the mean value of SA20-amino molecules at the silicon dioxide surface was 12 × 10^8^ cm^−2^, whereas on the bare graphene surface this mean number was 13 × 10^8^ cm^−2^. There was also no statistical difference between the number of molecules recovered from graphene and silicon dioxide when they were exposed with SA20-amino without previous thionine treatment of the surfaces.

Many of the graphene-based biosensors are produced in the FET (field effect transistor) configuration. In this architecture, graphene is on top of a silicon dioxide substrate, which acts as a dielectric layer between graphene and the highly-doped silicon. However, regarding graphene-based biosensors it is desirable that aptamers attach preferably on the surface of graphene, which is responsible for the sensing activity, rather on the silicon dioxide. It is clearly demonstrated by the results presented in this work that this is indeed the case if proper and matched aptamer and surface modifications are introduced.

In summary, we have developed and applied a systematic method for the analysis of aptamer-functionalized graphene and silicon dioxide samples using qPCR. In an unprecedented way, direct aptamer quantification of a functionalized 2D material was demonstrated, using an easy, robust, reproducible and direct methodology. By this new method, it was possible to compare different functionalization strategies, in two different materials, demonstrating that the combined use of thionine and SA20-amino presented better results over SA20-pyrene. In addition, it was demonstrated directly and quantitatively, by comparison of results for bare SiO_2_/Si substrates and SiO_2_/Si substrates with graphene, that pyrene-moieties have specific affinity to graphene, because of the stable π-stacking across the basal plane.

This novel tool has wide potential to be applied to any materials as long as the target functionalization is a DNA-based molecule. This technique can be readily and independently used to evaluate the efficiency of different functionalization strategies and to compare different methods and materials. More importantly, this approach is also a powerful tool to be applied in the biosensor production quality control.

## Supplementary information

Supplementary Figure 1
