# Supplementary material for: Real-time PCR for direct aptamer quantification on functionalized graphene surfaces
Source: Sci Rep. 2019 Dec 17;9:19311. doi: 10.1038/s41598-019-55892-3 (PMC6917711; doi:10.1038/s41598-019-55892-3)
Supplement: Supplementary file 1 — Supplementary Figure 1 [file 41598_2019_55892_MOESM1_ESM.docx]

**Real-time** **PCR for direct aptamer quantification on functionalized graphene surfaces**

Viviane C. F. dos Santos^1,2 *^, Nathalie B. F. Almeida^1,2^, Thiago A. S. L. de Sousa^1^, Eduardo N. D. Araujo^3^, Antero S. R. de Andrade^2^ & Flávio Plentz^1^

^1^ Departamento de Física, ICEx, Universidade Federal de Minas Gerais, Avenida Presidente Antônio Carlos 6627, Belo Horizonte, CEP 31270-901, Brazil

^2^ Centro de Desenvolvimento da Tecnologia Nuclear (CDTN), Avenida Presidente Antônio Carlos 6627, Campus Pampulha da Universidade Federal de Minas Gerais, Belo Horizonte, CEP 31270-901, Brazil

^3^ Departamento de Física, CCE, Universidade Federal de Viçosa, Avenida Peter Henry Rolfs, s/n, Viçosa CEP 36570-900, Brazil

* Corresponding author

1 2 3 M.W. 5 6 7 8


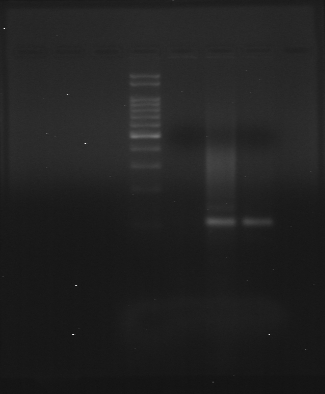


750

1000

200

250

100

150

50

**Figure 1.** **Representative results obtained using qPCR primer sets in conventional and qPCR. (a)** 2% typical agarose gel stained with ethidium bromide showing the amplification of the sequence of approximately 56 bp, referent of SA20 aptamer at the end of the PCR process stained with ethidium bromide showing molecular weight (M.W.), negative template control (NTC, lane 5), positive control – with 10 ng of SA20 aptamer (lane 6) and a SA20-pyrene functionalized graphene (lane 7), lane 1, 2, 3 and 8 were not used, so these lanes were cropped, and omitted in the figure 1 of the main text. Besides this, in the main text figure color was inverted in order to facilitate the results visualization. This assay was done in order to check PCR efficiency for primer sets used in qPCR assays.
